# TAS-116, a Well-Tolerated Hsp90 Inhibitor, Prevents the Activation of the NLRP3 Inflammasome in Human Retinal Pigment Epithelial Cells

**DOI:** 10.3390/ijms22094875

**Published:** 2021-05-05

**Authors:** Sofia Ranta-aho, Niina Piippo, Eveliina Korhonen, Kai Kaarniranta, Maria Hytti, Anu Kauppinen

**Affiliations:** 1 School of Pharmacy, University of Eastern Finland, 70211 Kuopio, Finland; niina.piippo@uef.fi (N.P.); eveliina.korhonen@uef.fi (E.K.); maria.hytti@uef.fi (M.H.); 2 Department of Clinical Chemistry, Helsinki University Hospital, University of Helsinki, 00290 Helsinki, Finland; 3 Department of Ophthalmology, Institute of Clinical Medicine, University of Eastern Finland, 70211 Kuopio, Finland; kai.kaarniranta@kuh.fi; 4 Department of Ophthalmology, Kuopio University Hospital, 70211 Kuopio, Finland

**Keywords:** NLRP3, Hsp90, TAS-116, age-related macular degeneration, retinal pigment epithelium

## Abstract

Chronic inflammation has been associated with several chronic diseases, such as age-related macular degeneration (AMD). The NLRP3 inflammasome is a central proinflammatory signaling complex that triggers caspase-1 activation leading to the maturation of IL-1β. We have previously shown that the inhibition of the chaperone protein, Hsp90, prevents NLRP3 activation in human retinal pigment epithelial (RPE) cells; these are cells which play a central role in the pathogenesis of AMD. In that study, we used a well-known Hsp90 inhibitor geldanamycin, but it cannot be used as a therapy due to its adverse effects, including ocular toxicity. Here, we have tested the effects of a novel Hsp90 inhibitor, TAS-116, on NLRP3 activation using geldanamycin as a reference compound. Using our existing protocol, inflammasome activation was induced in IL-1α-primed ARPE-19 cells with the proteasome and autophagy inhibitors MG-132 and bafilomycin A1, respectively. Intracellular caspase-1 activity was determined using a commercial caspase-1 activity kit and the FLICA assay. The levels of IL-1β were measured from cell culture medium samples by ELISA. Cell viability was monitored by the 3-(4,5-dimethylthiazol-2-yl)-2,5-diphenyltetrazolium bromide (MTT) test and lactate dehydrogenase (LDH) measurements. Our findings show that TAS-116 could prevent the activation of caspase-1, subsequently reducing the release of mature IL-1β. TAS-116 has a better *in vitro* therapeutic index than geldanamycin. In summary, TAS-116 appears to be a well-tolerated Hsp90 inhibitor, with the capability to prevent the activation of the NLRP3 inflammasome in human RPE cells.

## 1. Introduction

Inflammasomes are intracellular signaling complexes. The most extensively studied inflammasome contains a member of the NLR family, pyrin domain containing 3 (NLRP3) receptor, an apoptosis-associated speck-like protein containing a CARD (ASC), and caspase-1 proteins [1]. The assembly of the NLRP3 inflammasome activates caspase-1, which is needed for cleaving the proinflammatory cytokines IL-1β and IL-18 into their active forms, as well as for activating a cell death pathway called pyroptosis [2]. The NLRP3 inflammasome is a central regulator of inflammation and its activation has been associated with several age-related diseases, such as Alzheimer’s disease, atherosclerosis, Parkinson’s disease, and age-related macular degeneration (AMD) [1,3,4,5,6,7,8,9,10].

The activation of the NLRP3 inflammasome is a stringently regulated two-step process [1]. A priming signal, which commonly is transmitted through Toll-like receptors (TLRs) or cytokine receptors, induces the production of NLRP3 and pro-IL-1β by activating the transcription factor, nuclear factor kappa B (NF-кB) [1]. Thereafter, an activation signal leads to the oligomerization of NLRP3 molecules and the subsequent maturation of IL-1β and/or IL-18 through the activation of the caspase-1 enzyme [2]. In age-related macular degeneration (AMD), several examples of activators of the NLRP3 inflammasome have been identified, e.g., drusen and lipofuscin components, cathepsin B leaking from damaged lysosomes, and oxidative stress [9,11,12,13]. 

Mayor et al. used THP-1 cells and demonstrated that Heat shock protein (Hsp)90 is a crucial chaperone, protecting NLRP3 from destruction while keeping it intact but ready to be activated [14]. We have recently shown that the inhibition of Hsp90 by geldanamycin could prevent the NLRP3 activation-dependent IL-1β release from human retinal pigment epithelium (RPE) cells [15]. Some Hsp90 inhibitors have been developed and tested especially for cancer therapy [16]. There are pathogenic conditions where aberrant Hsp90 client proteins are thought to have a crucial role, e.g., in many neurodegenerative and aggregation diseases; therefore, Hsp90 inhibitors are an interesting option in treating those diseases as well [17]. 17-AAG, a synthetic derivative of geldanamycin, has shown advantageous properties, such as anti-inflammatory effects in murine models of endotoxin-induced uveitis, retinitis pigmentosa, and inherited retinal degeneration [18,19,20]. Thus, limited efficacy and poor solubility have limited further trials with 17-AAG [16]. Despite the positive results, when more potent Hsp90 inhibitors were examined in clinical trials, several serious adverse effects were observed; the most notable of these included ocular toxicity accompanied with visual disturbances [21,22,23,24]. 

Japanese researchers have recently developed 4-(1H-pyrazolo[3,4-b]pyridine-1-yl)benzamide, abbreviated to TAS-116, that is a selective inhibitor of cytosolic Hsp90α/β [25]. Unlike other Hsp90 inhibitors, such as 17-AAG, 17-DMAG, NVP-AUY922, BIIB021, or SNX-2112, TAS-116 does not inhibit other Hsp90 paralogs, e.g., GRP94 in the endoplasmic reticulum or TRAP1 in mitochondria [25]. Moreover, interactions between cytochrome P450 enzymes and TAS-116 are minimal [25]. Orally administered TAS-116 has not induced photoreceptor injuries in rats [25]. Additionally, TAS-116 was claimed to be well tolerated by human RPE cells [25]. In a phase I clinical trial in patients with gastrointestinal stromal tumor, TAS-116′s most common side effects were gastrointestinal disorders, increased serum creatinine levels, and reversible eye disorders [26].

In AMD, which is the leading cause of blindness in developed countries, NLRP3 is abundant in the retina, and IL-1β levels are high in the vitreous body [10,27,28]. We and others, have shown that the NLRP3 inflammasome is activated in cells of the retinal pigment epithelium (RPE) [8,11,12]. The RPE is a post-mitotic cell monolayer located between the retina and Bruch’s membrane [29]. It maintains the functionality of the photoreceptors and the homeostasis of the retina. In AMD, RPE degeneration is associated with increased oxidative stress, dysfunctional cellular clearance of damaged macromolecules, and inflammation [30]. Several investigations using cultured cells or in animal models of AMD, have shown that this inflammation can be alleviated by silencing NLRP3 or inhibiting caspase-1 [3,8,10,11,12,13,31,32]. Downregulation of the NLRP3 pathway has also been reported to improve the viability and the morphology of stressed RPE; therefore the regulation of NLRP3 via Hsp90 inhibition is a potential target to treat AMD, a disease that urgently needs new treatment options. [10,11,31].

In this study, we have examined the effects of TAS-116 on NLRP3 inflammasome activation and cell viability in human RPE cells, and compared them to the effects of geldanamycin. Our present data indicate that, similarly to geldanamycin, TAS-116 is able to reduce the caspase-1-dependent secretion of IL-1β from human RPE cells, while exhibiting a better tolerability profile than geldanamycin. 

## 2. Results

### 2.1. TAS-116 Is Better Tolerated by RPE Cells than Geldanamycin

**Figure 1:**
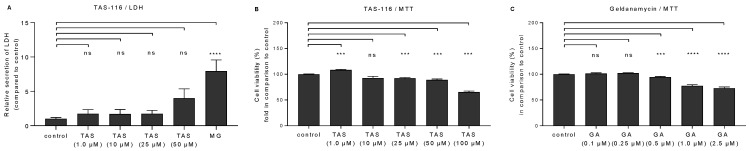
Cell viability upon exposure of ARPE-19 cells to TAS-116 or geldanamycin. Cells were treated for 48 h with the indicated TAS-116 (TAS) or geldanamycin (GA) concentrations. LDH release from TAS-treated cells was measured from cell culture medium samples and compared to the values of untreated control cells, which were set to a value of 1 (**A**). Cells exposed to MG-132 (MG) served as a positive control. MTT results of TAS-116 (**B**) or geldanamycin-treated cells (**C**) were compared to untreated control cells, the viability of which was set to 100%. Data are combined from two (**B**) or three (**A**,**C**) independent experiments with four parallel samples in each group. All results are presented as mean  ±  SEM. *** *p* < 0.001, **** *p*  <  0.0001, ns = nonsignificant, Mann–Whitney U test.

Since cytotoxicity is the major issue limiting the use of Hsp90 inhibitors [16], we applied two different methods to examine the viability of TAS-116-treated ARPE-19 cells. As the data from the lactate-dehydrogenase (LDH) assay indicate, TAS-116 concentrations from 1 µM to 25 µM caused only a minor increase in LDH levels (Figure 1A). A concentration of 50 µM showed a trend towards increased LDH release, although the difference to untreated control cells remained statistically non-significant (*p* = 0.13). Subsequently, MTT data were used to calculate cell viability (Figure 1B). When compared to untreated cells, a TAS-116 concentration of 100 µM caused a reduction of over 20% in cell viability, whereas geldanamycin reduced cell viability to below 80% at the concentration of 1.0 µM, (Figure 1C).

### 2.2. TAS-116 Reduces the Secretion of IL-1β and IL-8 from RPE Cells with Dysfunctional Intracellular Clearance

**Figure 2:**
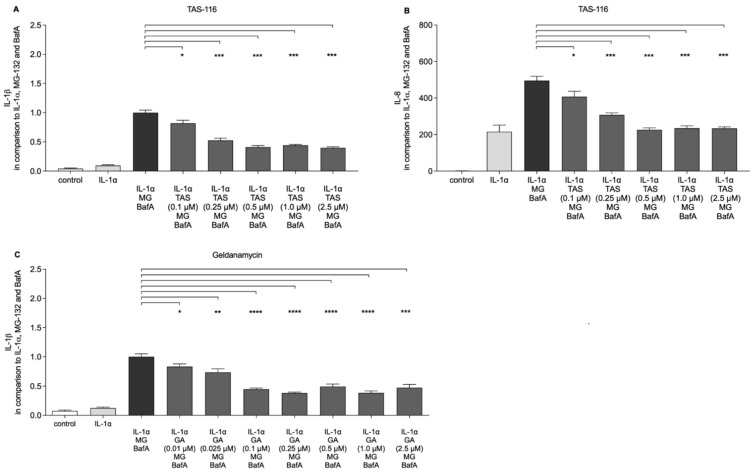
The effect of TAS-116 (TAS) on the release of IL-1β (**A**) and IL-8 (**B**), and the effect of geldanamycin (GA) on the release of IL-1β (**C**). IL-1α-primed RPE cells were exposed concurrently to TAS or GA and MG-132 (MG), and 24 h later to Bafilomycin A1 (BafA). IL-1β release from TAS- or GA-treated cells was measured from cell culture medium samples and compared to the values in the IL-1α + MG + BafA group, which was set to a value of 1. Data are combined from two to three independent experiments with four parallel samples in each group and are presented as mean  ±  SEM. * *p*  <  0.05, ** *p*  <  0.01, *** *p* < 0.001 **** *p*  <  0.0001, Mann–Whitney U test.

We have previously shown that disturbances in proteasomal and autophagy clearance promote ROS production and further activate NLRP3 inflammasome signaling in human RPE cells [3,15]. Since NLRP3 activation is known to depend on two distinct signals [32,33], we first primed the cells with IL-1α, as previously described [10,15]. Thereafter, the proteasome inhibitor MG-132 and lysosome neutralizer bafilomycin A1 (BafA) provided the activation signal to the NLRP3 inflammasome, which led to the secretion of IL-1β [3]. The exposure of RPE cells to 0.1–2.5 µM TAS-116 significantly reduced the secretion of IL-1β (Figure 2A). In addition, 0.5 µM TAS-116 reduced the secretion of IL-8 (Figure 2B). Since these TAS-116 concentrations were not cytotoxic (Figure 1A,B), the present data suggest that TAS-116 actively prevented the release of IL-1β and IL-8 from RPE cells with dysfunctional intracellular clearance, and furthermore, that the reduced interleukin levels did not result from cell death. In a comparison with TAS-116, we also measured the anti-inflammatory effect of geldanamycin (Figure 2C). A concentration of 0.01 µM geldanamycin was sufficient to significantly reduce the secretion of IL-1β in IL-1α, MG-132, and BafA-treated cells (Figure 2C).

### 2.3. TAS-116 Has a Higher Therapeutic Index than Geldanamycin In Vitro 

**Figure 3:**
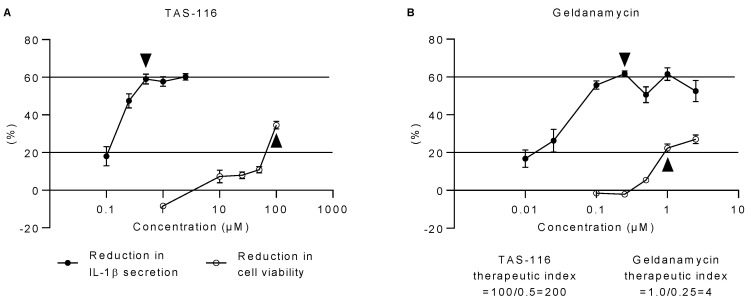
Therapeutic indices of TAS-116 (**A**) and geldanamycin (**B**). The therapeutic index is the ratio between toxic and therapeutic concentrations of a compound. When compared to primed RPE cells upon exposure to MG-132 and BafA, 0.5 µM TAS-116 and 0.25 µM geldanamycin concentrations, respectively, were sufficient to reduce the secretion of IL-1β by 60%. A TAS-116 concentration of 100 µM and a geldanamycin concentration of 1 µM reduced cell viability by over 20% when compared to untreated cells, as determined by the MTT assay. Arrowheads indicate the therapeutic (▼) and toxic concentrations (▲) of the compounds according to our predetermined cut-off points. Data are combined from two to three independent experiments with four parallel samples in each group and are presented as mean  ±  SEM.

The safety and potency of compounds can be combined to calculate their therapeutic index. The therapeutic index is a ratio between toxic and therapeutic concentrations. Our cut-off point for toxicity was the lowest concentration causing a reduction of over 20% in the cell viability. This follows the ISO 10993-5:2009 standard for Biological evaluation of medical devices, which states that over 80% cell viability is considered acceptable [34]. We defined the lowest concentration that reduced the secretion of IL-1β by 60% to be the therapeutic concentration, since this level of reduction appears to be the maximal anti-inflammatory response achievable with both TAS-116 and geldanamycin. The *in vitro* therapeutic index for TAS-116 was 200 (calculated by the Equation (1) presented in Section 4.5; Figure 3A,B), while for geldanamycin, it was only 4. This means that the TAS-116 concentration that reduced cell viability to below 80% was 200-fold higher than the concentration needed to achieve a 60% reduction in the secretion of IL-1β. In the case of geldanamycin, the concentration that reduced cell viability to below 80% was only 4 times higher than the concentration needed for a 60% reduction in the secretion of IL-1β. This clearly shows that TAS-116 exhibits a better tolerability profile than geldanamycin.

### 2.4. TAS-116 Prevents the Activation of Caspase-1

**Figure 4:**
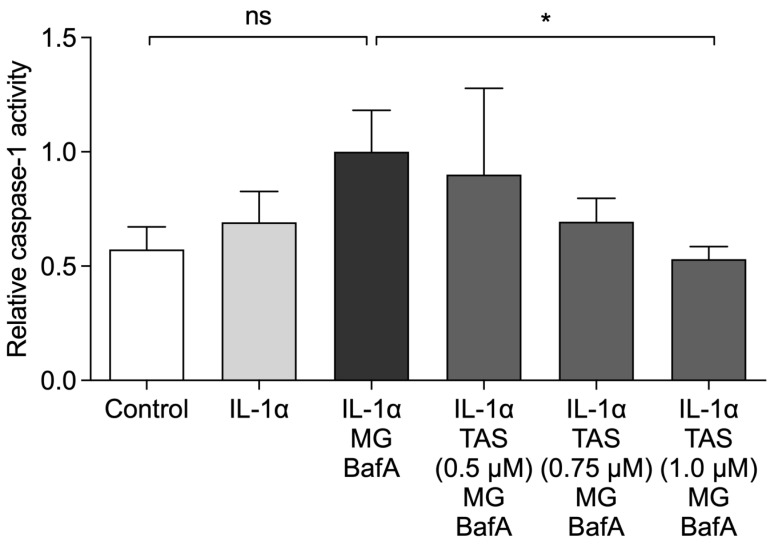
The enzymatic activity of caspase-1 was measured using a commercial kit. Results were normalized to the IL-1α + MG-132 (MG) + Bafilomycin A1 (BafA)-treated group. TAS-116 (TAS; 1.0 µM) reduced the enzymatic activity of caspase-1 in IL-1α-primed RPE cells exposed to MG + BafA. Data are combined from four independent experiments with two parallel samples in each group and are presented as mean  ±  SEM. * *p*  <  0.05, ns = not significant, Mann–Whitney U test.

**Figure 5:**
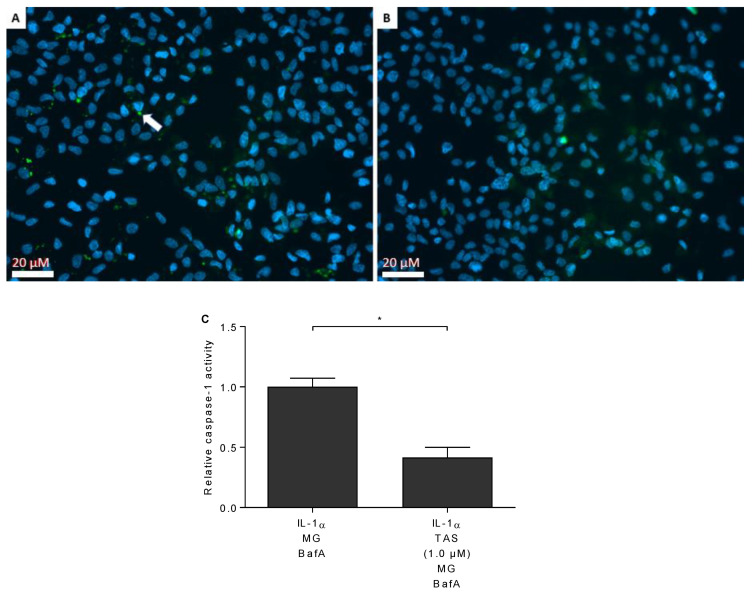
The measurement of caspase-1 activation using the FLICA assay. Nuclei were stained with Hoechst33342 (blue) and active caspase-1 with FLICA probe (green, white arrow) in IL-1α-primed RPE cells treated with MG-132 (MG) + Bafilomycin A1 (BafA; **A**), or with MG + BafA + TAS-116 (TAS; 1.0 µM; **B**). The amount of activated caspase-1 was compared to the number of nuclei and thereafter normalised to the IL-1α + MG + and BafA group (**C**). Images were taken at 20× magnification with one image per sample being shown. The data of the bar plot are combined from two individual experiments with two parallel samples per group and are presented as mean  ±  SEM. * *p*  <  0.05, Mann–Whitney U test.

Since the release of IL-1β is dependent on the cleavage of pro-IL-1β by caspase-1, we measured the activity of caspase-1 from ARPE-19 cell lysates. As shown in Figure 4, TAS-116 significantly reduced the caspase-1 activity when compared to primed RPE cells exposed to MG-132 + BafA without TAS-116. The effect of TAS-116 on caspase-1 activity was concentration-dependent (Figure 4). We further confirmed this finding by using the FLICA probe FAM-YVAD-FMK. Caspase-1 activation (green) was increased in primed cells upon exposure to MG-132 and BafA, whereas TAS-116 (1.0 µM) treatment reduced the activation of caspase-1 (Figure 5). 

### 2.5. TAS-116 Has No Effect on the Levels of Hsp90 or Hsp70 Increased by the Decline in Intracellular Clearance

**Figure 6:**
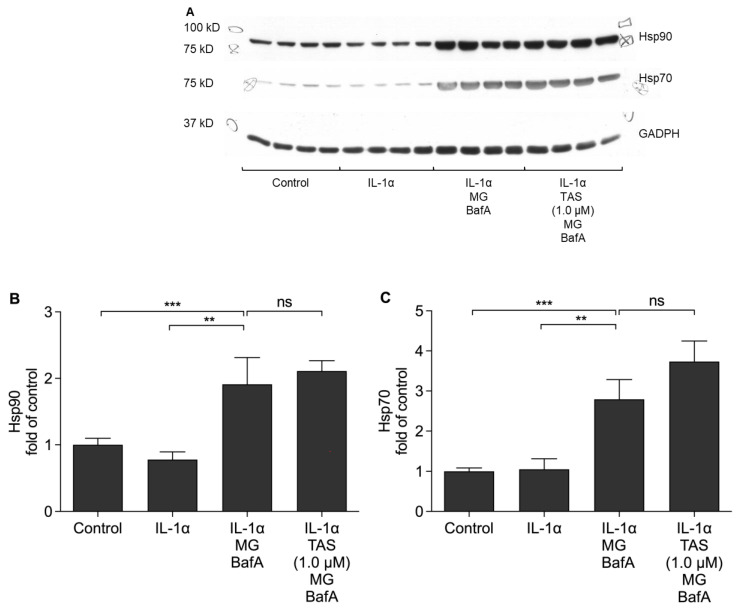
Representative images of protein levels of Hsp90 and Hsp70 in cell lysates determined using the Western blot technique (**A**). Quantified protein levels of Hsp90 (**B**) and Hsp70 (**C**). IL-1α-primed RPE cells treated with MG-132 (MG) + Bafilomycin A1 (BafA) and TAS-116 (TAS) if indicated. Data are combined from two independent experiments with four parallel samples in each group per experiment. Results are presented as mean  ±  SEM. ** *p*  <  0.01, *** *p* < 0.001, ns = nonsignificant, Mann–Whitney U test.

We have previously shown that Hsp90 inhibition with geldanamycin promotes the removal of NLRP3 [15]. In order to examine whether Hsp90 would be removed along with NLRP3, we measured intracellular protein levels of Hsp90 using the Western blot method. The protein levels of Hsp90 were increased following the cellular stress induced by MG-132 and BafA (Figure 6A,B). TAS-116 had no effect on the Hsp90 protein levels (Figure 6A,B). Since Hsp90 inhibition is known to induce the production of other Hsp proteins [35,36], we tested whether TAS-116 could influence the levels of Hsp70. Similarly to Hsp90, an exposure of RPE cells to MG-132 and BafA increased the Hsp70 levels (Figure 6A,C). TAS-116 treatment further increased the levels of Hsp70 but the effect was not statistically significant (*p* = 0.23).

## 3. Discussion

The degeneration of RPE cells plays a major role in the development of AMD [30]. Various stress factors lead to the activation of the NLRP3 inflammasome in RPE cells, promoting inflammation and stress [8,12,13]. Prevention of NLRP3 inflammasome activation has been shown to increase the viability of the stressed RPE [10,11,31]. This forms the basis why new drug candidates, such as natural polyphenols, which prevent IL-1β secretion by inhibiting the function of NLRP3 or other inflammasome components, have lately been under scrutiny in relation to immunological, neurological, and metabolic diseases [37,38]. Our research interest was focused on the anti-inflammatory effect of Hsp90 inhibitors, since previous therapeutic applications of Hsp90 inhibitors have been beset by ocular toxicity [21,22,23,24]. For example, in the study of Sessa et al. (2013), 23% of the patients who received the Hsp90 inhibitor AUY922, suffered from night blindness [23]. In addition to adverse effects, the development of Hsp90 inhibitors has struggled with finding the optimal balance between effectiveness and tolerability [16]. In the therapy of retinal diseases, even high drug concentrations should not cause ocular toxicity, while low doses should suffice to evoke a therapeutic response. 

From a pharmacological point of view, one of the most important factors is the ratio between the therapeutic effect and toxicity. In the present *in vitro* study, TAS-116 exhibited a 50 times higher therapeutic index than geldanamycin, a compound which was examined in our previous study [15]. The therapeutic index is usually derived from *in vivo* results with the formula LD50/ED50 (LD50 = lethal dose for 50% of population, ED50 = minimum effective dose for 50% of population). There are no widely accepted criteria for what would represent a drug with a narrow therapeutic index [39], but a rule of thumb is that an index lower than 3 or 4 is narrow [40]. The *in vitro* therapeutic index can be used as a predictive value for the *in vivo* index [41]. A limitation of the *in vitro* therapeutic index is the lack of a universally accepted calculation approach, and the fact that there are no guidelines for estimating the correlation between *in vitro* and *in vivo* derived therapeutic indexes. However, one fact about the *in vitro* therapeutic index is clear: the higher the better.

Hsp90α and Hsp90β are the two major mammalian Hsp90 isoforms and they share great structural and functional identity [42]. Generally, Hsp90α is inducible and Hsp90β is constitutively expressed, and TAS-116 is capable of inhibiting both isoforms (Hsp90α Ki 34.7 nmol/L, Hsp90β Ki 21.3 nmol/L) [25,42]. We did not analyze Hsp90 isoforms in the present study, but a recent study suggests that inflammasome signaling can be regulated only by inhibiting Hsp90α [43]. In the present study, we have also concentrated only on RPE, and in future studies, photoreceptors need more detailed examination. That is justified since Hsp90 inhibitors can be directly toxic to photoreceptor cells [44]. In the publication of Ohkubo et al. (2015), two-weeks treatment with TAS-116 did not cause any visible retinal damage on rats although the peak concentration in the retina was 4 µM, 8 times higher than the lowest therapeutic concentration in our article [25]. Therefore, expectations for safety are high, which also applies with respect to new studies.

Despite utilizing the same cell line (ARPE-19), our absolute viability values in response to TAS-116 exposure differed from the results of Suzuki et al. [45]. We observed only a 20% reduction in cell viability with TAS-116 at a 100 µM concentration, while TAS-116 at a much lower level, 0.625 µM, evoked this level of cytotoxicity in the work of Suzuki et al. [45]. A similar phenomenon was observed in their analysis of the toxicity of 17-AAG, a semi-synthetic analog of geldanamycin [45]. The 17-AAG concentration causing over 20% toxicity in their experiment was 0.313 µM, while our cells tolerated a concentration of up to 1 µM of the more toxic geldanamycin before reaching the same limit [45]. However, this kind of inter-laboratory variation between cell lines is a common phenomenon. Despite the different values, our data showed a similar trend with the findings of Suzuki et al. Thus, both research groups suggest that RPE cells tolerate TAS-116 better than other Hsp90 inhibitors [45].

Our previous study demonstrated that geldanamycin treatment prevented NLRP3 inflammasome activation [15]. Our present data showing the reduced release of IL-1β, suggest that TAS-116 functions in a similar manner. This, supported by other publications, indicates that the anti-inflammatory effect of Hsp90 inhibitors is related to the activation of NLRP3 [14,15,46,47,48]. All these publications have shown that Hsp90 inhibition reduces the secretion of IL-1β, which requires the inflammasome-associated action of caspase-1 for its cleavage and maturation. Correspondingly, Hsp90 inhibition has also been shown to result in reduced caspase-1 activity [14,15,48]. Mayor et al. and Li et al. applied immunoprecipitation techniques and demonstrated that Hsp90 physically interacts with NLRP3 in human embryonic kidney 293T and murine microglial BV2 cells, respectively [14,48]. In the present study, we used ARPE-19 cells that as undifferentiated line cells, serve as an initial experimental model for the anti-inflammatory effects of TAS-116. In subsequent studies, we need to confirm our findings using primary human RPE cells and a suitable animal model.

Zuo et al. treated mice with 17-AAG after an experimental subarachnoid hemorrhage and found that the resulting Hsp90 inhibition reduced the protein level of Hsp90 in brain tissue [47]. We did not observe any change in the protein levels of Hsp90 in ARPE-19 cells after the inhibition of Hsp90 (Figure 6B). One possible explanation is that Hp90 is degraded by either proteasomal clearance or autophagy, i.e., processes which were blocked in our cell model [49,50]. On the other hand, Beck et al. conducted an *in vitro* study with K562 cells and reported that Hsp90 inhibition with 17-AAG did not induce the degradation of Hsp90 even when proteasomal clearance was functional [50]. In comparison to the study of Beck et al. [50], our cell type was different. Furthermore, when comparing *in vitro* and *in vivo* results, cell-cell interactions should be taken into account. Inflammation can induce the production of Hsp90, and the anti-inflammatory effect of Hsp90 inhibition could suppress this upregulation [51]. Therefore, in the study of Zuo et al., the reduced Hsp90 protein levels may have resulted from reduced synthesis instead of increased degradation [47].

In addition to the anti-inflammatory effect of Hsp90 inhibitors, the induction of other Hsp chaperones has been recognized as a beneficial effect in experimental disease models of neuronal aggregation diseases, such as Alzheimer’s, Parkinson’s, or Huntington’s diseases [52]. In relation to AMD, it has been shown that increased intracellular Hsp70 levels are able to protect cells from the damage evoked by oxidative stress [53,54]. In addition to the NLRP3-related anti-inflammatory effect, upregulation of Hsp70 could be another beneficial effect of Hsp90 inhibitors in the treatment of AMD. In the present study, the production of Hsp70 was induced by both MG-132 and BafA exposure, and TAS-116 showed a trend for further inducing the production of Hsp70. Batulan et al. and Yanagitani et al. have shown, using geldanamycin and TAS-116, respectively, that Hsp90 inhibition results in the production of Hsp70 [26,35]. The increased production of Hsp70 and Hsp27 after Hsp90 inhibition has also been observed in RPE cells [36]. In our present study, this effect may have been masked due to the upregulation of Hsp70 induced by MG-132 and BafA.

Due to its anti-inflammatory properties, lower retinal toxicity, increased specificity only to the cytosolic Hsp90, and minimal interactions with cytochrome P450 enzymes, TAS-116 appears to represent a promising drug candidate, and superior to the other Hsp90 inhibitors described in the literature. A challenge to be resolved in the therapy of ocular diseases is that, similarly to many other small molecules, the retinal half-life of TAS-116 is short [25,55]. As shown in a phase I clinical trial to treat patients with gastrointestinal stromal tumor, the reversible therapy of eye disorders should also be possible [26]. Adverse effects are usually dose-dependent, and dosing depends on the disease. For example, high methotrexate doses are a suitable treatment option for cancer, while lower doses are used as an immunosuppressant in rheumatic diseases [56]. Similarly, the dosing protocol needed to treat AMD would probably differ from that administered in a clinical trial treating patients with a gastrointestinal stromal tumor, i.e., the selection of the appropriate dose needs careful optimization. Evidently, *in vivo* animal experiments will be the next step in determining whether TAS-116 has an effective and safe dosing window for the treatment of retinal disorders. Our results showing a high *in vitro* therapeutic index, indicate that it should be possible to determine a TAS-116 concentration which has a good anti-inflammatory effect without generating excessive adverse effects. 

## 4. Materials and Methods 

### 4.1. Cells and Stimulations

The experiments were carried out with ARPE-19 cells (American Type Culture Collection, Manassas, VA, USA), which is a human-derived RPE cell line [57]. The passage numbers of the cells ranged from 28 to 38. In the experiments, the cells were placed in 12-well plates (Costar, Corning incorporated, Kennebunk, ME, USA) or on 8 chamber Lab-Tek chamber slides (Nunc Lab-Tek II Chamber Slide; Thermo Fisher Scientific, Rochester, NY, USA) at the concentration of 200,000 cells/mL. Cells were cultured in a humidified 5% CO_2_ atmosphere at 37 °C in DMEM with the nutrient mixture F-12 1:1 mixture (Life Technologies, Carlsbad, CA, USA), 100 U/mL penicillin, 100 µg/mL streptomycin (Life Technologies, Grand Island, NY, USA), 2 mM L-glutamine (Life Technologies, Paisley, UK), and 10% fetal bovine serum (FBS; Hyclone, Logan, UT, USA). After incubation for 3 or 2 days on cell culture plates or chamber slides, respectively, confluent wells were washed with serum-free medium, which was also used throughout the experiments.

In the cell viability assays, the cells were treated with TAS-116 (Active Biochem, Hongkong, China) at the indicated concentrations for 48 h. In the other experiments, the cells were primed with 4 ng/mL recombinant human IL-1α (R&D systems, Minneapolis, MN, USA) for 24 h. TAS-116 was added simultaneously with 5 µM MG-132 (Calbiochem, San Diego, CA, USA) and incubated for 24 h. Subsequently, 50 nM Bafilomycin A1 (BafA; Sigma-Aldrich, Saint Louis, MO, USA) was added into the indicated wells for another 24 h. MG-132, TAS-116, and geldanamycin were all dissolved in DMSO (Sigma-Aldrich, Saint Louis, MO, USA). Cell cultures differing only by the absence or presence of Hsp90 inhibitor had equal concentrations of DMSO. 

### 4.2. Sample Collection

Medium samples were centrifuged (380× *g*, 10 min), and supernatants were transferred into clean microtubes and stored at −20 °C until analyzed. The cells were washed with Dulbecco’s phosphate buffered saline (DPBS; Life Technologies, Paisley, UK). In the caspase-1 activity assay, the cells were collected into fresh DPBS and centrifuged (16,060× *g*, 1 min), subsequently the, supernatants were discarded and pellets were stored at −20 °C. Cells used in the Western blot measurements were lysed with M-PER^®^ solution according to the manufacturer’s instructions (Sigma-Aldrich, Rockford, IL, USA). The lysate was centrifuged (16,060× *g*, 1 min), supernatants were transferred into clean tubes, and the tubes were stored at −80 °C until analyzed.

### 4.3. Cell Viability Assays

The 3-(4,5-dimethylthiazol-2-yl)-2,5-diphenyltetrazolium bromide (MTT) assay was used to determine the metabolic activity of the cells. The assay was performed as previously described [58]. To determine the integrity of the cellular membranes, lactate dehydrogenase (LDH) levels were measured from medium samples using the commercial CytoTox 96^®^ Non-Radioactive Cytotoxicity assay according to the manufacturer’s protocol (Promega Corporation, Madison, WI, USA).

### 4.4. ELISA Measurements

IL-1β and IL-8 were measured from cell culture medium samples using BD OptEIA^TM^ assays and following the manufacturer’s protocol (San Diego, CA, USA). Where needed, samples were diluted in Assay Diluent (BD OptEIA^TM^, San Diego, CA, USA). A commercial TMB substrate solution (BD OptEIA^TM^, San Diego, CA, USA) was used for the IL-1β and IL-8 measurements. OD values were measured at a wavelength 450 nm with a reference wavelength of 655 nm using a spectrophotometer (Bio-Rad Model 550 with the Microplate Manager 5.2 programme; Bio-Rad Laboratories, Inc., Hercules, CA, USA)

### 4.5. Calculation of the Therapeutic Index

The therapeutic index is derived from the relative reduction in viability and the relative effectiveness.

(1) $$\mathrm{Therapeutic}\;\mathrm{index}=\frac{C_{reduction\;in\;viability}\;\geq \;20\%}{C_{reduction\;in\;secretion\;of\;IL-1\beta }\;\geq \;60\%},$$

To calculate relative toxicity, we used data obtained using the MTT assay. Single OD values were normalized to controls, which were set to 100%.

To calculate the reduction in the secretion of IL-1β, we used data obtained using IL-1β ELISA. Since the secretion of IL-1β increases after primed cells are treated with MG-132 and BafA [15], this group was used as a control and was set to 100%. C stands for concentration.

### 4.6. Caspase-1 Activity Assays

The activity of caspase-1 was measured from cell lysates using a commercial kit (Caspase-1 Colorimetric Assay: R&D systems, Minneapolis, MN, USA) according to the manufacturer’s instructions. Caspase-1 activity was further analyzed using the Pyroptosis/Caspase-1 Assay (FLICA; ImmunoChemistry, Bloomington, MN, USA) in cultured cells according to the manufacturer’s instructions. Briefly, cells grown on 8-well chamber slides were stained following the manufacturer’s protocol, were fixed with 4% PFA, stained with Hoechst33342 (NucBlue^TM^, Life Technologies, Eugene, OR, USA), and mounted with Mowiol (Sigma, St. Louis, MO, USA). Images, one per well, two per treatment group, were taken on the next day and adjusted with the same settings. Manually counted green caspase spots were compared to the number of cell nuclei. The experiment was repeated twice. In total, 1 342 IL-1α + MG-132 + BafA and 1 457 IL-1α + MG-132 +TAS-116 (1.0 µM) + BafA-treated cells were counted.

### 4.7. Western Blot Assays

Cell lysates containing 20 µg protein were mixed with 4x NuPage LDS sample buffer (Invitrogen, Carlsbad, CA, USA) and β-mercaptoethanol (Fluka, Neu Ulm, Switzerland). The final concentration of β-mercaptoethanol was 5%. Samples were incubated at 95 °C for 4 min and then loaded onto 10% SDS-PAGE gels. Proteins were wet-blotted overnight from gels onto nitrocellulose membranes (Amersham, Pittsburgh, PA, USA). The membranes were blocked for 2 h at room temperature (RT) in Tris-buffered saline (TBS; 50 mM Tris, 150 mM NaCl) containing 5% fat-free milk powder (Valio, Lapinlahti, Finland) and 0.1% Tween-20 (Sigma-Aldrich, Saint Louis, MO, USA).

Mouse monoclonal Hsp70 antibody (Enzo ADI-SPA-810) was diluted 1:5000 in 0.3% Tween-20/phosphate buffered saline (PBS; 137 mM NaCl), and the membranes were incubated for 1 h at RT. Thereafter, the membranes were washed three times with 0.3% Tween-20/PBS and incubated for 1 h at RT with horseradish peroxidase-conjugated anti-mouse IgG antibody (NA931, GE Healthcare, Little Chalfont, Buckinghamshire, UK) diluted 1:40,000 in washing buffer containing 3% milk powder. Rat monoclonal Hsp90 antibody (Abcam ADI-SPA-83-SF, Abcam, Cambridge, MA, USA) was diluted 1:5000 in 0.3% Tween-20/PBS containing 0.5% BSA, and the membranes were incubated for 2 h at RT. The washes were performed similarly to those for Hsp70. Anti-rat IgG antibody (NA935Y, GE Healthcare, Little Chalfont, Buckinghamshire, UK) served as the secondary antibody, and was diluted 1:20,000 in 0.3% Tween-20/PBS containing 3% milk powder. Mouse monoclonal anti-glyceraldehyde-3-phosphate dehydrogenase (GAPDH; Abcam ab8245, Abcam, Cambridge, MA, USA) was diluted 1:15,000 in 0.3% Tween-20/PBS. The same buffer was used for the washes and dilution of the secondary antibody. Membranes were incubated overnight at +4 °C. The secondary antibody, anti-mouse IgG antibody (NA931, GE Healthcare, Little Chalfont, Buckinghamshire, UK) was diluted 1:12,000. Membranes were incubated with the secondary antibody for 2 h at RT. The membranes were washed again, and Immobilon Western Chemiluminescent HRP Substrate (Millipore, Billerica, MA, USA) was added to the membranes. Super Rx medical X-ray films (Fuji Corporation, Tokyo, Japan) were used to detect the chemiluminescence, and the band intensities were analyzed with the ImageJ software (U. S. National Institutes of Health, Bethesda, MD, USA; http://rsb.info.nih.gov/ij, accessed on 4 May 2021). The intensities of the bands were normalized to GAPDH.

### 4.8. Data and Statistical Analyses

Mann-Whitney U test was applied for pairwise statistical analyses performed using the GraphPad Prism 8.4.0 (GraphPad Software Inc., San Diego, CA, USA). *p*-values 0.05 or less were considered statistically significant. All independent experiments containing 4–6 parallel samples per group were repeated at least three times if not otherwise stated.

## 5. Conclusions

From the group of currently available Hsp90 inhibitors, TAS-116 seems to be one of the promising drug candidates, furthermore it also seems that it will be active in the ocular environment. Owing to its high *in vitro* therapeutic index, it should be possible to find a TAS-116 concentration achieving a good anti-inflammatory effect in the eye without causing disturbing adverse effects.

## Data Availability

Data available on request from the authors.
